# The combined effect of Covid-19 and neighbourhood deprivation on two dimensions of subjective well-being: Empirical evidence from England

**DOI:** 10.1371/journal.pone.0255156

**Published:** 2021-07-23

**Authors:** Franco Bonomi Bezzo, Laura Silva, Maarten van Ham

**Affiliations:** 1 La Statale, University of Milan, Milan, Italy; 2 Department of Sociology, Sciences Po, Paris, France; 3 CREST, ENSAE Paris, Palaiseau, France; 4 Department of Urbanism, Faculty of Architecture and the Built Environment, Delft University of Technology, Delft, The Netherlands; 5 School of Geography and Sustainable Development, University of St Andrews, Scotland, United Kingdom; Sun Yat-sen University, CHINA

## Abstract

**Objectives:**

The Covid-19 pandemic is hitting societies hard, and people living in disadvantaged circumstances are among the most affected. We investigate the combined effects of the Covid-19 crisis and living in a deprived neighbourhood on two dimensions of subjective well-being: hedonic (i.e. mental health) and evaluative (i.e. life satisfaction) subjective well-being.

**Methods:**

We use longitudinal data from the Understanding Society UK panel. We combine data gathered in the main survey between 2015 and 2019 with very recent data from the Covid-19 online survey between April and July 2020. Leveraging a sample of nearly 9,600 English individuals, we first run a set of cross-sectional OLS regressions to analyse changes over time in the relationship between neighbourhood deprivation and subjective well-being. Then, as our main model of interest, we use a fixed effect difference-in-differences model to provide more robust evidence.

**Results:**

Since the beginning of the crisis, both levels of hedonic and evaluative well-being have decreased as a result of the pandemic and lockdown. However, for those living in more deprived neighbourhoods the level of hedonic well-being decreased more than for those living in better areas. We found no such difference for evaluative well-being.

**Conclusion:**

Our results highlight the importance of reducing neighbourhood inequalities as the spatial clustering of disadvantages has increased by the pandemic.

## Introduction

The Covid-19 pandemic is an unprecedented event in modern history, and an event with massive implications in terms of health and well-being inequalities [1]. Individual psychological and health-related outcomes have been significantly affected by both the concrete risk of getting the virus, and the policies adopted by governments to stop its spread. Among these, lockdown measures in particular, which have limited people’s movements beyond their house and local area, have been found to crucially affect individuals’ mental health and well-being [2–6].

The Covid-19 crisis has hit individuals in different situations in different ways. In this study we focus on the effect of the level of socio-economic deprivation of the residential neighbourhood. The relationship between neighbourhood deprivation and well-being has been widely debated [7]. Many have argued that individuals living in neighbourhoods characterised by higher levels of socio-economic deprivation tend to display lower levels of subjective well-being as compared to those living in less deprived areas [8–10]. Some, tough, have not been able to detect a clear relationship between neighbourhood socio-economic deprivation and individual well-being [11].

The spread of the pandemic offers new opportunities to investigate the relationship between neighbourhood deprivation and well-being. Lockdown measures throughout the pandemic have indeed forced individuals to limit their movements and, thus, likely led them to experience more deeply the characteristics of their own residential neighbourhoods. There is growing evidence that Covid-19 has exacerbated existing spatial inequalities. Already in April 2020, few weeks after the outburst of the disease, an article in The Guardian remarked how Covid-19 had significantly worsened the situation of those who already lived in the poorest urban areas such as Seine-Saint-Denis, the most deprived neighbourhood in Paris [12]. Considering all this, this paper focusses on the question whether Covid-19 and the related lockdown measures have affected the relationship between the quality of the neighbourhood where individuals live and their well-being.

Aligning ourselves with the main well-being literature [13–17], we consider two different dimensions of well-being, hedonic and evaluative. The former is the result of positive and negative feelings, which may be derived from day-to-day conditions and experiences such as one’s immediate health state. The latter is the result of the evaluation of one’s own life overall and tends to involve an element of comparison between the individual’s situation and hir (for gender neutrality, throughout the text we will exclusively use the pronouns ze/hir) reference group. While the first tends to focus more on the short-term and is often considered a good proxy of mental health, the second one is usually related to long-term goals and opportunities and tends to proxy life-satisfaction. While they are crucially related [18], it must be stressed that it is important to accurately report and specify which of the two dimension we are referring to as such distinction has been deemed to be important on both empirical and theoretical grounds [19].

A number of works have analysed the extent to which the Covid-19 pandemic has influenced individuals’ mental health and well-being. Mental health, or alternatively, as we define it, hedonic well-being, has decreased as a consequence of the pandemic using the Understanding Society UK panel [20, 21]. Such an effect is particularly pronounced in April when the UK was in a full lockdown, while the effect diminished in the following months. Authors also tested the effect of Covid-19 on other health-related outcomes, such as smoking and drinking [22], or loneliness [23]. Evaluative well-being has received, instead, less attention so far. From the scarce literature we know that in Germany, individuals’ life satisfaction, or evaluative well-being, has decreased between March and May 2020 [24]. In the US, individuals have displayed lower levels in both dimensions of subjective well-being, hedonic and evaluative, after the pandemic has started [25]. Various studies have assessed the effect of the pandemic on the well-being of individuals by socio-economic status [25], gender [26, 27] or ethnicity [28]. Area-based characteristics, such as neighbourhood deprivation, have received limited attention. As an exception, a study on cross-sectional data from Wales found that during the Covid-19 pandemic, neighbourhood socio-economic deprivation has increased the negative effect of the pandemic on well-being and mental health [29].

In this work, we aim to shed additional light on the effect of Covid-19 on individuals’ mental health and well-being, and focus in particular on neighbourhood deprivation as a contextual factor that intervenes in this relationship. Our starting hypothesis is that individuals who live in more deprived neighbourhoods, which tend to be more densely populated with smaller houses and overall less desirable living conditions, have experienced the effect of the pandemic more severely than those living in less deprived areas. During the lockdown, people have not had the same opportunities to “escape” the neighbourhood as in “normal times”. Rather, they have been forced to spend more time at their residence, they could only move outside in the immediate surroundings of their home, and they have been more likely to have in person interactions only with their neighbours. Therefore, we expect neighbourhood deprivation to have had and inflating effect on an already unequal pre-existing situation. Furthermore, we also expect that the greatest drop in well-being has occurred during the months of the full lockdown (April and May covered in the survey), and that with time passing by this effect has come back to normality.

Across the multiple devastating consequences of Covid-19, including social and economic effects, the effects on mental health and well-being have been defined so far as “the elephant in the room” [30]. That’s why they have also been increasingly identified as a research priority [31]. In fact, uncovering potential ways to minimise the negative impact of the pandemic, especially on those who were already, even before Covid-19, in disadvantaged situations, is crucial to understand and reduce existing inequalities. This study provides three major contributions to the literature. First, to the best of our knowledge we are the first to analyse the combined effect of neighbourhood deprivation and the Covid-19 crisis on subjective well-being in the UK. Second, we disentangle this effect over two distinct but equally important dimensions of well-being, hedonic and evaluative well-being. Finally, exploiting the longitudinal nature of the dataset, we provide robust evidence by adopting a difference-in-differences strategy, which aims to increase causality in the explanation of the relationships.

## Materials and methods

### Data

We use data from Understanding Society, the UK Household Longitudinal Study (UKHLS), a nationally representative longitudinal study which began in 2009 and covers over 100,000 individuals from around 40,000 households in the UK [32]. Individuals from the main Understanding Society sample have been invited starting April 2020 to complete a one short web-survey a month. The survey is aimed at understanding the changing impact of the pandemic on the welfare of UK individuals, families and wider communities. In May 2020, participants who do not use the internet were surveyed by leveraging a telephone version of the questionnaire. Participants gave informed oral consent to take part in each wave of the study and were enrolled only after consent was provided. All survey procedures were approved by the Ethics Committee of the University of Essex. Data is available to researchers via the UK Data Service under special licence conditions for geocoded data. In our study we consider the four most recent yearly survey waves from 2015 to 2019 and the first four monthly Covid study waves from April to July 2020. Overall, across the UK, of those who took part in the latest wave available from the main survey (Wave 0, with individuals surveyed from 2017 to 2019), 46% completed the April Covid-19 survey. Response rates were similar amongst the surveys in May (48.5%), June (48.6%) and July (48.4%). Our initial entire sample (people aged 16 and above living in the UK) includes of 49,659 single observations. We use this information in combination with data on neighbourhood socio-economic status in England as provided by the UK Ministry of Housing, Communities and Local Government (MHCLG) [33]. To measure the degree of deprivation of the neighbourhood our respondents live in, we use the 2019 Index of Multiple Deprivation (IMD). We define the neighbourhood at the level of Lower Layer Super Output Areas (LSOAs, of which there are 32,482 in England). LSOAs are areas consistent in size and their boundaries do not change (unlike electoral wards), which makes them well suited for measuring the Index of Multiple Deprivation every five years and analysing how it changes over time. Each LSOA contains between 1,000 and 3,000 people.

We first selected individuals who live in England (N = 38,614), and we retained those who have at least one observation before the pandemic and one observation after (N = 15,736). Finally, our empirical sample is composed of those people who have not changed neighbourhood across the observation period (i.e. since 2015). For the cross-sectional analysis we consider all waves from 2015 to July 2020 and our empirical sample is therefore composed of 11,952 (Hedonic) and 11,931 (Evaluative) individual observations with a non-missing value. For the difference-in-differences model, we restrict our sample from 2015 to just the first two Covid-19 waves to analyse the more pure effects of the lockdown, which leads to 9,622 (hedonic) and 9,615 (evaluative) individual observations with a non-missing value. In fact, in the UK, lockdown measures were strictly put in place at the end of March and started being progressively released at the beginning of May, when individuals were first allowed again to go out once a day for exercising. We thus are interested in the specific role of the neighbourhood in shaping the influence of the pandemic on well-being during this period, as we expect the neighbourhood dimension to be less relevant once lockdown restrictions are released.

### Measures

#### Neighbourhood socio-economic deprivation

To measure neighbourhood deprivation we use the score dimension of the 2019 Index of Multiple Deprivation (IMD). The IMD is composed of 9 weighted domains: Income (.225), Employment (.225), Education Skills and Training (.135), Health and Disability (.135), Crime (.093), Barriers to Housing and Services (.093), Living Environment (.093). The variable is computed such that the higher the score, the more deprived is the neighbourhood. In other words, a negative value of the coefficient for the deprivation variable in our analysis would suggest that living in a more deprived neighbourhood is associated with a decrease of subjective well-being.

#### Hedonic well-being

The indicator of hedonic well-being we use as our key outcome variable is derived from the General Health Questionnaire (GHQ). It is one of the most renowned measures of subjective well-being [34], made up of a 12-item scale designed to assess symptoms such as anxiety, insomnia, happiness and severe depression using questions such as: ‘Have you recently lost much sleep over worry?’; ‘Have you recently felt constantly under strain?’ and ‘Have you recently been able to enjoy your normal day-to-day activities?’. There are four possible answers, two of them are positive (i.e. ‘more than usual’) and two of them are negative (i.e. ‘less than usual’). Responses are computed in a score ranging from 0 (best psychological well-being) to 36 (worst psychological well-being) for each participant. To simplify comparison with measures of evaluative well-being, we reverse this variable so that individuals are scored from 0 (worst hedonic well-being) to 36 (best hedonic well-being).

#### Evaluative well-being

Our measure of evaluative well-being consists in a self‐reported measure of life satisfaction, which is related to a person’s thoughts about hir life. The exact wording of the question is: ‘All things considered, how satisfied or dissatisfied are you with your life overall using a 1–7 scale? 1 = very dissatisfied, …, 7 = very satisfied.’ While we have information on hedonic well-being at each Covid-19 wave, we have information on evaluative well-being only in the 2^nd^ (May) and 4^th^ (July) waves.

### Statistical analysis

The empirical analysis is composed of a) 12 (six for each well-being measure) cross-sectional models in which we look at the effect of neighbourhood deprivation on well-being in each wave adding controls at each step according to a forward selection approach; and b) six longitudinal fixed-effect models. We estimate all models using sampling weights [35] and robust standard errors clustered at LSOA level using STATA 16.0. Our decision of clustering at LSOA level is motivated by the fact that, as we identify the neighbourhood at LSOA level, we want to avoid within-cluster correlation biases at the treatment level.

In the first part of the empirical analysis, our initial cross-sectional model is the basic model with only the IMD. We then add a number of individual characteristics: ethnicity (either white or not), age and gender (asked as binary, male or female), a dummy for the presence or not of previous health problems, perceived financial security (low, medium, high), employment status (employed, self-employed, unemployed), the possibility to work from home (never, sometimes, always), and a variable which controls whether the individual lives with a partner or not. We proceed including household characteristics: household size (which ranges from 1 to 14), equivalised household income, number of children under 16 in the household (which ranges from 0 to 7), the type of tenure (owner, private renter, social renter, other). We finally control for two contextual variables; whether the individual lives within an urban context or not, and the geographical region (which are 9 macro regions, e.g. West Midlands).

The second part of the empirical analysis concerns the difference-in-differences approach. The latter is particularly well suited for our analysis because it allows exploiting the variability of deprivation across English neighbourhoods around the sharp discontinuity created by the unexpected lockdown measures introduced to face the spread of Covid-19.

Specifically, we clarify here our basic specification and describe it as follows:

$$y_{i,t}=\alpha +\beta_{1}Post_{i,t}+\beta_{2}(Nhb_{i}*Post_{i,t})+\gamma_{i}+\delta_{t}+\eta_{t}+u_{i,t}$$

where the dependent variable, yi,t, is the level of subjective well-being reported by individual i at time t. Posti,t is a dummy for the post-Covid period and Nhbi is the level of neighbourhood deprivation experienced by individual i. Included are also *γ_i_*, individual fixed effects, *δ_t_*, wave fixed effects and *η_t_*, month fixed effect. In the full specifications with then add also time-varying covariates.

Using a fixed-effect modelling structure allows us to take into account all time-invariant individual characteristics. Therefore, in our models we first include the interaction term (Nhb_i_*Post_i,t_) without controlling for anything else. To mitigate concerns related to the fact that the nature of our data and strategy forces us to compare pre and post Covid-19 periods of non-equivalent length, we add in a second stage wave and month controls. Finally, we also control for the remaining time-variant individual characteristics. In this way, we can study the overall mean effect of the Covid-19 crisis on the correlation between neighbourhood deprivation and individual subjective well-being across different specifications.

## Results

Table 1 contains descriptive statistics of our sample. In the first and second columns it reports respectively the mean values for the 0 wave of Understanding Society (which corresponds to the survey wave running between 2017–2019) and for the May wave of the Covid-19 survey when individuals had been in full lockdown for two months. Columns 3 to 7 report the mean values in May 2020 for each Neighbourhood Deprivation quintile (the 1^st^ being the least deprived and the 5^th^ the most deprived).

**Table 1:** Descriptive statistics, mean(sd).

	Wave -1	Wave 2	Wave 2				
	All	All	1st Quintile	2nd Quintile	3rd Quintile	4th Quintile	5th Quintile
Evaluative Well-being (std)	5.197 (1.445)	4.753 (1.581)	5.000 (1.504)	4.962 (1.515)	4.752 (1.554)	4.601 (1.631)	4.508 (1.626)
Hedonic Well-being (std)	25.820 (5.464)	24.546 (6.217)	25.689 (5.149)	25.225 (5.403)	24.989 (6.021)	24.014 (6.508)	23.109 (7.188)
Neighbourhood Deprivation	-0.043 (0.987)	0.089 (1.071)	-0.991 (0.114)	-0.661 (0.093)	-0.289 (0.127)	0.316 (0.225)	1.731 (0.841)
Age	51.345 (16.599)	52.585 (17.743)	55.732 (17.387)	54.213 (17.199)	54.348 (17.691)	52.391 (17.553)	47.267 (17.546)
Female	0.540 (0.498)	0.518 (0.500)	0.514 (0.500)	0.512 (0.500)	0.491 (0.500)	0.554 (0.497)	0.517 (0.500)
Low Educ	0.051 (0.221)	0.067 (0.249)	0.032 (0.176)	0.060 (0.238)	0.073 (0.260)	0.054 (0.226)	0.107 (0.309)
Medium Educ	0.410 (0.492)	0.448 (0.497)	0.357 (0.479)	0.416 (0.493)	0.443 (0.497)	0.477 (0.500)	0.529 (0.499)
High Educ	0.450 (0.497)	0.385 (0.487)	0.520 (0.500)	0.451 (0.498)	0.405 (0.491)	0.352 (0.478)	0.231 (0.422)
Other	0.085 (0.279)	0.096 (0.295)	0.086 (0.281)	0.072 (0.259)	0.079 (0.270)	0.114 (0.318)	0.123 (0.329)
White	0.948 (0.221)	0.913 (0.282)	0.962 (0.190)	0.954 (0.209)	0.948 (0.221)	0.925 (0.264)	0.796 (0.403)
Underlying health condition	0.362 (0.480)	0.469 (0.499)	0.432 (0.496)	0.457 (0.498)	0.466 (0.499)	0.504 (0.500)	0.481 (0.500)
Low Financial Security	0.062 (0.240)	0.056 (0.230)	0.023 (0.150)	0.028 (0.166)	0.061 (0.240)	0.050 (0.218)	0.108 (0.311)
Medium Financial Security	0.179 (0.383)	0.165 (0.371)	0.111 (0.314)	0.128 (0.335)	0.151 (0.358)	0.193 (0.395)	0.228 (0.420)
High Financial Security	0.756 (0.429)	0.761 (0.427)	0.856 (0.351)	0.829 (0.377)	0.764 (0.424)	0.745 (0.436)	0.636 (0.481)
Never Work at Home	0.395 (0.489)	0.274 (0.446)	0.226 (0.418)	0.231 (0.422)	0.247 (0.431)	0.286 (0.452)	0.363 (0.481)
Sometimes Work at Home	0.148 (0.356)	0.098 (0.297)	0.109 (0.312)	0.111 (0.315)	0.115 (0.320)	0.093 (0.291)	0.066 (0.248)
Always Work at Home	0.041 (0.199)	0.202 (0.402)	0.231 (0.422)	0.254 (0.436)	0.217 (0.412)	0.204 (0.403)	0.121 (0.327)
Living with partner	0.688 (0.463)	0.633 (0.482)	0.722 (0.448)	0.702 (0.457)	0.665 (0.472)	0.614 (0.487)	0.493 (0.500)
Unemployed	0.396 (0.489)	0.440 (0.496)	0.446 (0.497)	0.431 (0.495)	0.427 (0.495)	0.430 (0.495)	0.461 (0.499)
Self-employed	0.075 (0.264)	0.083 (0.277)	0.087 (0.282)	0.099 (0.299)	0.101 (0.302)	0.092 (0.290)	0.044 (0.205)
Employee	0.487 (0.500)	0.469 (0.499)	0.460 (0.499)	0.465 (0.499)	0.464 (0.499)	0.473 (0.499)	0.479 (0.500)
Numbers of children	0.398 (0.811)	0.397 (0.806)	0.324 (0.710)	0.389 (0.796)	0.373 (0.754)	0.416 (0.838)	0.469 (0.893)
HH size	2.811 (1.318)	2.794 (1.328)	2.729 (1.206)	2.782 (1.253)	2.689 (1.240)	2.732 (1.353)	3.006 (1.503)
Living in Urban Area	0.234 (0.423)	0.225 (0.417)	0.237 (0.426)	0.323 (0.468)	0.330 (0.470)	0.207 (0.405)	0.056 (0.231)
North East	0.766 (0.423)	0.769 (0.422)	0.754 (0.431)	0.670 (0.470)	0.664 (0.473)	0.786 (0.410)	0.939 (0.240)
North West	0.048 (0.214)	0.051 (0.220)	0.048 (0.215)	0.025 (0.157)	0.028 (0.165)	0.060 (0.237)	0.088 (0.283)
Yorkshire and the Humber	0.125 (0.331)	0.133 (0.340)	0.103 (0.304)	0.113 (0.316)	0.147 (0.354)	0.100 (0.301)	0.194 (0.396)
East Midlands	0.101 (0.301)	0.104 (0.306)	0.065 (0.247)	0.085 (0.280)	0.099 (0.299)	0.098 (0.297)	0.163 (0.369)
West Midlands	0.091 (0.287)	0.088 (0.283)	0.084 (0.277)	0.124 (0.330)	0.100 (0.300)	0.077 (0.267)	0.060 (0.238)
East of England	0.103 (0.303)	0.105 (0.306)	0.085 (0.279)	0.104 (0.306)	0.098 (0.298)	0.095 (0.293)	0.137 (0.344)
London	0.124 (0.329)	0.122 (0.328)	0.117 (0.321)	0.133 (0.340)	0.167 (0.373)	0.140 (0.347)	0.063 (0.243)
South East	0.125 (0.331)	0.129 (0.335)	0.092 (0.289)	0.098 (0.297)	0.099 (0.299)	0.166 (0.373)	0.176 (0.381)
South West	0.178 (0.383)	0.173 (0.379)	0.298 (0.457)	0.226 (0.418)	0.160 (0.366)	0.143 (0.350)	0.066 (0.249)
Living in Urban Area	0.106 (0.308)	0.094 (0.292)	0.109 (0.312)	0.091 (0.288)	0.102 (0.303)	0.122 (0.327)	0.053 (0.225)
Household earnings, low	0.243 (0.429)	0.384 (0.486)	0.300 (0.459)	0.337 (0.473)	0.356 (0.479)	0.442 (0.497)	0.466 (0.499)
Household earnings, med.	0.367 (0.482)	0.164 (0.370)	0.190 (0.392)	0.194 (0.396)	0.159 (0.366)	0.155 (0.362)	0.128 (0.334)
Household earnings, high	0.389 (0.487)	0.135 (0.342)	0.202 (0.401)	0.166 (0.372)	0.150 (0.357)	0.109 (0.312)	0.064 (0.245)
Observations	7982	6018	1310	1281	1257	1199	971

mean coefficients; sd in parentheses.

Note: weighted.

With respect to our outcome variables we can see that individuals overall tend to report lower levels of well-being in May 2020 than in 2018 and there is a gradient in neighbourhood deprivation with decreasing mean values across the deprivation quintiles. People living in the more deprived neighbourhoods are younger on average than those living in the least deprived neighbourhoods. Overall, the figures of all our covariates, both at the individual and the household level, confirm what we would expect. The more deprived the neighbourhood, the lower the proportion of high educated individuals, the lower the proportion of white people, and the higher the proportion of people living in urban areas. Interestingly, while there is a difference across deprivation quintiles in terms of financial security and earnings, there does not seem to be a difference in terms of employment status. On the one hand, the household size and the number of children is larger in more deprived neighbourhoods than in less deprived ones but, on the other hand, the proportion of people living with a partner is much lower. Finally, those living in more deprived areas tend to work less from home and to report more underlying health conditions than those living in less deprived areas.

**Fig 1:**
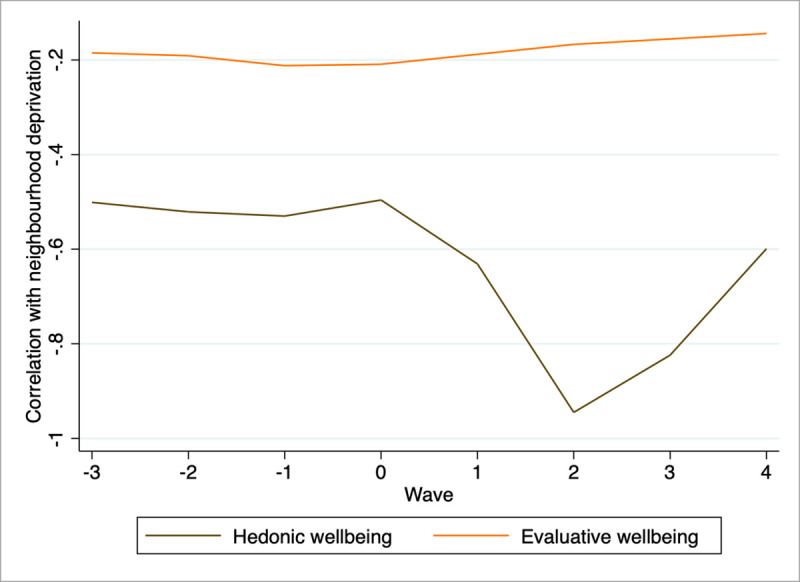
Neighbourhood correlation with hedonic and evaluative wellbeing, base model, OLS cross-section by wave. Note: Weighted results, all coefficients statistically significant at p<0.01. For evaluative wellbeing, results are estimated only in wave 2 and 4. Authors computation on Understanding Society.

Fig 1 shows the correlation between neighbourhood deprivation and our two wellbeing measures without adding any covariate. Fig 1 shows that in wave 2 (i.e. May), when we expect the effect of the prolonged lockdown to be at its peak, neighbourhood deprivation has a much stronger negative correlation with hedonic well-being compared to the pre-pandemic period. On the other hand, in wave 4 (i.e.July), once the lockdown has been released, the correlation starts to come back to pre-lockdown values. This is not the case for evaluative well-being, as the negative relationship with neighbourhood deprivation keeps on decreasing (less negative, thereore upward slope of the curve) after the outburst of the pandemic until wave 4.

S1–S6 Tables present the base models (from which we took the values reported in Fig 1) with only the neighbourhood deprivation indicator as main covariate and intermediate models.

It is striking that when we add individual and household characteristics (S1–S4 Tables), we observe that thispatterns remains remarkably consistent. In the full model, when everything else is taken into account (Tables 2 and 3), the most interesting result is again the pattern of the effect of the neighbourhood deprivation coefficients on hedonic well-being. The coefficient passes from being not significant in the pre-Covid-19 period to become strongly significant and large after around two months of lockdown (wave 2) and then starts to decrease again toward pre-pandemic values when lockdown measures are released.

**Table 2:** Hedonic Well-being, full model OLS cross-section by wave.

	Pre-Covid-19				Covid-19			
VARIABLES	-3	-2	-1	0	1	2	3	4
**Neighbourhood deprivation**	**-0.027**	**0.071**	**0.054**	**0.073**	**-0.050**	-0.363**	**-0.229**	**-0.181**
	**(0.077)**	**(0.075)**	**(0.079)**	**(0.057)**	**(0.121)**	**(0.143)**	**(0.140)**	**(0.131)**
	**Individual**							
Gender (female)	-1.021***	-0.817***	-0.927***	-0.844***	-1.749***	-1.599***	-1.509***	-1.158***
	(0.114)	(0.117)	(0.122)	(0.096)	(0.200)	(0.229)	(0.231)	(0.214)
Age	0.045***	0.047***	0.058***	0.057***	0.067***	0.062***	0.064***	0.056***
	(0.006)	(0.006)	(0.006)	(0.005)	(0.011)	(0.012)	(0.012)	(0.010)
Ethnicity (non-white)	0.535**	0.533**	0.530**	0.461***	0.273	0.664	0.612	1.255**
	(0.245)	(0.247)	(0.254)	(0.169)	(0.514)	(0.564)	(0.521)	(0.495)
Medium education	0.237	0.017	-0.352	-0.336	-0.656	-0.741	-0.881	-0.125
	(0.287)	(0.301)	(0.312)	(0.234)	(0.563)	(0.546)	(0.631)	(0.602)
High education	-0.015	-0.287	-0.664**	-0.438*	-1.115**	-1.119**	-1.132*	-0.622
	(0.290)	(0.306)	(0.313)	(0.235)	(0.555)	(0.532)	(0.625)	(0.583)
Other education	0.464	-0.159	-0.525	-0.335	-0.954	-1.209	-1.124	0.014
	(0.333)	(0.347)	(0.373)	(0.277)	(0.703)	(0.744)	(0.745)	(0.634)
Mid financial security	3.627***	3.875***	4.465***	3.776***	4.195***	4.162***	4.797***	6.194***
	(0.452)	(0.440)	(0.447)	(0.304)	(0.789)	(0.886)	(0.728)	(0.819)
High financial security	5.561***	5.902***	6.530***	6.177***	7.100***	7.360***	6.529***	9.053***
	(0.431)	(0.413)	(0.425)	(0.287)	(0.732)	(0.827)	(0.683)	(0.749)
Underlying health problem	-2.155***	-2.025***	-2.224***	-2.295***	-1.106***	-0.878***	-1.578***	-0.915***
	(0.137)	(0.133)	(0.135)	(0.107)	(0.216)	(0.241)	(0.232)	(0.219)
Self-employed	0.715***	0.342	0.122	0.676***	0.821	-0.341	0.379	1.432
	(0.274)	(0.261)	(0.271)	(0.225)	(0.994)	(0.802)	(0.894)	(0.966)
Employee	0.516**	0.352	-0.202	0.139	0.595	-0.484	0.175	1.317
	(0.236)	(0.221)	(0.245)	(0.190)	(0.986)	(0.765)	(0.859)	(0.951)
Can work from home: sometime	-0.287	-0.371**	-0.405**	-0.338**	-0.109	-0.266	-0.587	-0.498
	(0.176)	(0.170)	(0.176)	(0.144)	(0.337)	(0.373)	(0.448)	(0.305)
Can work from home: always	-0.016	0.155	-0.115	-0.266	-0.605**	-0.730**	-0.574*	-1.063***
	(0.280)	(0.281)	(0.304)	(0.245)	(0.271)	(0.303)	(0.326)	(0.276)
Living with a partner	0.231	0.076	-0.099	0.197	0.478*	0.415	0.569*	0.228
	(0.162)	(0.157)	(0.163)	(0.129)	(0.281)	(0.285)	(0.329)	(0.283)
	**Household**							
Private rent	0.327	-0.144	0.321	0.139	-0.287	-2.487	0.146	0.073
	(0.275)	(0.298)	(0.286)	(0.244)	(1.025)	(1.818)	(0.519)	(0.443)
Social rent	-0.613**	-1.235***	-0.939***	-0.910***	-0.510	-0.529	-0.397	-0.462
	(0.262)	(0.269)	(0.285)	(0.214)	(0.405)	(0.375)	(0.470)	(0.418)
Other tenure	-2.256	1.889**	-1.447	-0.515	-0.022	-0.365	0.771	-0.580
	(1.922)	(0.744)	(1.613)	(0.916)	(1.146)	(1.052)	(0.969)	(1.114)
Number of children in the hh	-0.070	-0.013	0.129	0.129*	-0.351**	-0.386**	-0.661***	-0.154
	(0.099)	(0.093)	(0.102)	(0.074)	(0.177)	(0.187)	(0.246)	(0.197)
Household size	0.133*	0.156**	0.113	0.027	0.126	0.341**	0.393**	0.116
	(0.074)	(0.071)	(0.077)	(0.048)	(0.112)	(0.146)	(0.163)	(0.135)
Household earnings, med.	-0.139	-0.385**	0.053	-0.206	-0.179	-0.343	-0.188	-0.112
	(0.164)	(0.174)	(0.180)	(0.140)	(0.263)	(0.313)	(0.309)	(0.254)
Household earnings, high	-0.131	-0.237	0.230	-0.204	-0.055	-0.206	0.127	-0.005
	(0.169)	(0.173)	(0.181)	(0.149)	(0.283)	(0.296)	(0.317)	(0.331)
	**Context**							
Living in an urban area	-0.250*	-0.253*	-0.182	-0.315***	-0.546**	-0.346	-1.036***	-0.546**
	(0.128)	(0.132)	(0.142)	(0.116)	(0.263)	(0.276)	(0.252)	(0.225)
North West	-0.023	0.577*	-0.014	-0.146	0.050	0.012	0.329	0.382
	(0.326)	(0.338)	(0.345)	(0.275)	(0.599)	(0.736)	(0.653)	(0.525)
Yorkshire and Humber	0.103	0.365	0.017	-0.040	0.273	0.543	0.154	1.023**
	(0.253)	(0.263)	(0.266)	(0.200)	(0.474)	(0.549)	(0.508)	(0.418)
East Midlands	0.094	0.349	-0.127	0.006	-0.006	0.271	0.527	0.736*
	(0.262)	(0.263)	(0.275)	(0.207)	(0.478)	(0.549)	(0.491)	(0.421)
West midlands	0.019	0.458*	0.102	0.119	-0.454	0.046	-0.310	-0.707
	(0.256)	(0.276)	(0.263)	(0.212)	(0.502)	(0.518)	(0.502)	(0.590)
East of England	-0.510*	-0.078	-0.197	-0.072	0.063	-0.215	0.635	0.273
	(0.263)	(0.270)	(0.271)	(0.206)	(0.468)	(0.515)	(0.501)	(0.548)
London	-0.007	0.070	-0.019	-0.064	-0.055	0.415	-0.132	0.493
	(0.252)	(0.266)	(0.268)	(0.201)	(0.506)	(0.524)	(0.549)	(0.442)
South East	-0.068	0.336	-0.014	0.007	0.024	0.422	0.427	0.413
	(0.227)	(0.237)	(0.238)	(0.184)	(0.430)	(0.455)	(0.441)	(0.431)
South West	0.105	0.461*	0.039	-0.333	-0.337	0.358	-0.075	0.531
	(0.247)	(0.261)	(0.265)	(0.214)	(0.534)	(0.511)	(0.458)	(0.472)
Constant	19.648***	19.445***	19.036***	19.215***	17.390***	17.328***	17.825***	14.649***
	(0.753)	(0.757)	(0.806)	(0.565)	(1.559)	(1.612)	(1.470)	(1.473)
Observations	9,298	9,292	9,219	10,924	8,090	7,475	7,132	6,991
R-squared	0.140	0.163	0.195	0.188	0.186	0.196	0.179	0.224

Robust standard errors in parentheses

*** p<0.01

** p<0.05

* p<0.1; Reference categories: Education (Low), Employment (Unemployed), Can work from home (Never), Financial security (Low), Household earnings (Low),Tenure (Owned), Region (North East). Household size goes from 1 to 14, number of children in the household ranges 0 to 7. Weighted results.

**Table 3:** Evaluative Well-being, full model OLS cross-section by wave.

	Pre-Covid-19				Covid-19	
VARIABLES	-3	-2	-1	0	2	4
Neighbourhood deprivation	-0.034*	-0.012	-0.049**	-0.030**	-0.032	-0.047
	(0.019)	(0.020)	(0.020)	(0.014)	(0.032)	(0.035)
	**Individual**					
Gender (female)	0.019	-0.004	0.097***	0.014	0.009	0.034
	(0.030)	(0.031)	(0.032)	(0.024)	(0.062)	(0.059)
Age	0.006***	0.006***	0.008***	0.006***	0.002	-0.000
	(0.002)	(0.002)	(0.002)	(0.001)	(0.003)	(0.003)
Ethnicity (non-white)	-0.037	-0.061	-0.080	-0.082*	0.091	-0.048
	(0.065)	(0.064)	(0.068)	(0.042)	(0.111)	(0.123)
Medium education	-0.018	-0.067	-0.062	-0.031	-0.056	-0.065
	(0.081)	(0.081)	(0.089)	(0.066)	(0.159)	(0.154)
High education	-0.044	-0.062	-0.030	0.018	0.063	0.006
	(0.082)	(0.081)	(0.089)	(0.067)	(0.161)	(0.154)
Other education	-0.021	0.040	-0.015	-0.052	-0.197	-0.084
	(0.099)	(0.095)	(0.102)	(0.078)	(0.223)	(0.193)
Mid financial security	0.699***	0.865***	0.796***	0.816***	0.873***	0.970***
	(0.107)	(0.096)	(0.096)	(0.066)	(0.150)	(0.149)
High financial security	1.444***	1.566***	1.534***	1.597***	1.452***	1.735***
	(0.101)	(0.089)	(0.090)	(0.062)	(0.151)	(0.139)
Underlying health problem	-0.430***	-0.473***	-0.466***	-0.481***	-0.179***	-0.225***
	(0.034)	(0.034)	(0.034)	(0.027)	(0.060)	(0.059)
Self-employed	-0.025	0.000	0.106	0.033	-0.038	0.376*
	(0.076)	(0.075)	(0.072)	(0.057)	(0.245)	(0.203)
Employee	0.022	-0.032	-0.080	-0.071	-0.114	0.374*
	(0.060)	(0.062)	(0.064)	(0.046)	(0.241)	(0.197)
Can work from home: sometime	-0.023	-0.002	-0.061	-0.008	-0.017	-0.189*
	(0.043)	(0.044)	(0.046)	(0.035)	(0.099)	(0.097)
Can work from home: always	-0.009	0.126*	-0.041	-0.013	-0.039	-0.091
	(0.070)	(0.072)	(0.076)	(0.061)	(0.074)	(0.079)
Living with a partner	0.200***	0.132***	0.096**	0.131***	0.254***	0.138*
	(0.041)	(0.041)	(0.043)	(0.032)	(0.073)	(0.071)
	**Household**					
Private rent	0.022	-0.050	-0.045	-0.049	-0.305	0.018
	(0.072)	(0.079)	(0.076)	(0.059)	(0.270)	(0.110)
Social rent	-0.250***	-0.353***	-0.268***	-0.262***	-0.171*	-0.094
	(0.068)	(0.068)	(0.069)	(0.053)	(0.100)	(0.120)
Other tenure	-0.711*	0.506***	0.353	0.155	-0.407**	-0.057
	(0.370)	(0.176)	(0.397)	(0.193)	(0.206)	(0.219)
Number of children in the hh	0.029	0.000	0.054**	0.029	0.010	0.044
	(0.023)	(0.025)	(0.025)	(0.019)	(0.052)	(0.054)
Household size	-0.000	0.020	0.022	-0.002	0.012	0.034
	(0.018)	(0.018)	(0.017)	(0.012)	(0.032)	(0.035)
Household earnings, med.	-0.041	-0.040	-0.011	0.016	-0.033	-0.072
	(0.042)	(0.044)	(0.045)	(0.035)	(0.078)	(0.083)
Household earnings, high	-0.005	0.006	0.025	0.086**	0.072	0.050
	(0.045)	(0.046)	(0.046)	(0.037)	(0.074)	(0.087)
	**Context**					
Living in an urban area	-0.056	-0.100***	-0.054	-0.035	-0.061	-0.157**
	(0.034)	(0.036)	(0.039)	(0.030)	(0.072)	(0.064)
North West	0.095	0.129	0.127	-0.018	0.364**	0.251*
	(0.087)	(0.088)	(0.088)	(0.070)	(0.156)	(0.151)
Yorkshire and Humber	0.054	0.054	0.130*	0.038	0.194	0.137
	(0.068)	(0.069)	(0.071)	(0.050)	(0.131)	(0.128)
East Midlands	0.067	0.046	0.079	0.103**	0.342**	0.125
	(0.068)	(0.072)	(0.073)	(0.052)	(0.137)	(0.134)
West midlands	0.014	0.087	0.126*	0.100*	0.222	0.045
	(0.071)	(0.074)	(0.075)	(0.054)	(0.153)	(0.129)
East of England	-0.014	-0.063	0.060	0.029	0.232*	0.150
	(0.069)	(0.073)	(0.074)	(0.051)	(0.135)	(0.141)
London	0.086	0.015	0.081	0.004	0.160	0.009
	(0.067)	(0.071)	(0.072)	(0.051)	(0.146)	(0.147)
South East	0.051	0.005	0.067	0.002	0.296**	0.090
	(0.061)	(0.066)	(0.065)	(0.047)	(0.115)	(0.125)
South West	0.082	0.028	0.097	0.049	0.186	0.015
	(0.066)	(0.072)	(0.072)	(0.053)	(0.135)	(0.140)
Constant	3.822***	3.756***	3.517***	3.557***	3.305***	3.123***
	(0.188)	(0.188)	(0.192)	(0.135)	(0.394)	(0.378)
Observations	9,320	9,313	9,261	10,974	7,500	7,016
R-squared	0.147	0.169	0.179	0.202	0.111	0.123

Robust standard errors in parentheses

*** p<0.01

** p<0.05

* p<0.1; Reference categories: Education (Low), Employment (Unemployed), Can work from home (Never), Financial security (Low), Household earnings (Low),Tenure (Owned), Region (North East). Household size goes from 1 to 14, number of children in the household ranges 0 to 7. Weighted results.

Moving then to our main model of interest, the difference-in-differences fixed effect model, Table 4 shows three different specifications for each of our well-being outcomes. In column (1) and (3), beside the time-invariant characteristics taken into account by the nature of the fixed-effect model, we control only for the Covid-19 dummy and its interaction with the deprivation variable. In columns (2) and (4) we additionally take into account month, wave and individual time-varying characteristics.

**Table 4:** Hedonic and Evaluative Well-being, Fixed-effects model, Difference-in-differences across the Covid-19 outburst.

	Hedonic		Evaluative	
VARIABLES	Plain	Full	Plain	Full
**Covid#stdIMDscore**	-0.227*	-0.223**	**0.0197**	**0.0141**
	**(0.121)**	**(0.104)**	**(0.033)**	**(0.0309)**
Age		-0.105		-0.0177
		(0.103)		(0.0293)
Medium Educ		-0.995		-0.0731
		(0.701)		(0.168)
High Educ		-1.663**		-0.0112
		(0.817)		(0.251)
Medium Financial Security		2.721***		0.477***
		(0.323)		(0.0712)
High Financial Security		3.958***		0.785***
		(0.343)		(0.0791)
Household earnings, med.		-0.0800		0.0181
		(0.139)		(0.0391)
Household earnings, high		0.105		0.0764*
		(0.144)		(0.0447)
Underlying health problem		-0.394***		-0.135***
		(0.114)		(0.0328)
Living with partner		0.612		0.205*
		(0.404)		(0.120)
Number of children in the hh		-0.443***		-0.0413
		(0.136)		(0.0416)
Household size		0.0691		0.00126
		(0.0924)		(0.0252)
Self-employed		0.504		0.0837
		(0.364)		(0.133)
Employee		1.012***		0.145
		(0.372)		(0.143)
Can work from home: sometime		-0.284		0.0150
		(0.231)		(0.0781)
Can work from home: always		-0.551**		0.0802
		(0.248)		(0.0747)
Covid	-1.260***		-0.449***	
	(0.0955)		(0.0331)	(.0694)
Constant	25.64***	28.26***	5.132***	5.163***
	(0.0306)	(5.395)	(0.00567)	(1.523)
Observations	51,459	51,459	43,511	43,511
Month fixed effects	NO	YES	NO	YES
Wave fixed effects	NO	YES	NO	YES
R-squared	0.025	0.072	0.027	0.055
Number of pidp	9,622	9,622	9,615	9,615

Robust standard errors in parentheses

*** p<0.01

** p<0.05

* p<0.1; The interaction is composed by the continuous standardised IMD and a dummy variable taking value 1 in the waves of full lockdown, namely 1 and 2 and 0 in waves -3 to 0; Reference categories: Education (Low), Employment (Unemployed), Can work from home (Never), Financial security (Low), Household earnings (Low), Household size goes from 1 to 14, number of children in the household ranges 0 to 7. Weighted results. Since ad hoc weights for longitudinal analysis combining multiple pre-Covid 19 and post-Covid 19 waves are not available, we align to the literature and use a combination of the available weights.

Looking at the effect of other time-varying individual and contextual characteristics may help in the interpretation of our results and of the mechanisms behind the two different trends for hedonic and evaluative wellbeing.

Higher educated individuals have suffered more from the pandemic in terms of hedonic well-being than lower educated individuals while they do not seem to suffer more in terms of evaluative wellbeing. The correlation between age and wellbeing remains positive and does not seem to be affected by the pandemic and its restrictions.

Focussing on employment characteristics, something to notice is also that, more than the employment status, what seems to matter are working conditions. For hedonic well-being, we see a negative and significant effect of working from home, something which is not mirrored in terms of evaluative well-being. This may reflect that individuals find it very stressful to work from home, both in terms of working hours and their inability to separate work from private life, although this does not have an impact on their long-term life satisfaction. The self-reported assessment of financial security is a crucial indicator. In fact, both in terms of evaluative and, to a much larger extent, hedonic well-being, individuals who feel more financially secure report much higher levels of well-being. Having an underlying health problem is negatively correlated with well-being, although it must be noticed that such negative effect is reduced once the health crisis starts (Tables 2 and 3), potentially as an effect of the comparisons of these individuals with those who are sick due to the pandemic and whose terrible conditions are reported constantly by daily news. It must be noticed that in our sample the percentage of respondents who reported having Covid-19 is below 1%, and because this is unlikely low, we do not control for that.

Living with a partner is associated with increased subjective well-being for both hedonic and evaluative well-being, and this association grows stronger during the lockdown waves. Having company at home is good for hedonic well-being. Interestingly, while overall the number of children at home has a negative effect on hedonic wellbeing, the cross-sectional results show that the number of children in the household changes from having a positive correlation before the pandemic outbreak to a negative one as soon as the pandemic starts for hedonic well-being. However, the same variable for evaluative well-being keeps a positive correlation, although not significant throughout the pandemic waves. A possible explanation for this is that while kids remain a source of long-term life satisfaction, in the short-term, and especially in lockdown conditions, they might cause parents higher stress and decrease their momentaneous well-being (hedonic).

The results are quite consistent across the different specifications and whatever we control for they all depict a consistent picture. In fact, on average, the pandemic has had the consequence of further decreasing the hedonic well-being of individuals living in more deprived neighbourhoods, while it has a non-significant effect on the relationship between neighbourhood deprivation and evaluative well-being.

In order to mitigate concerns related to the disproportionate bandwidths before and after the DID treatment event, we present five additional models that we believe strengthen our results. What significantly and importantly changes in these models is that we have tried to create synthethic yearly subsamples which allow us to run our analysis on yearly observations (S7 Table).

First, we restrict our sample to individuals who have just one observation before the pandemic and one observation after the pandemic, taking into account only those interviewed always in April OR May (model 1) or, just, in April (model 2) of each year (we have tried to run the model also on May alone, but the number of observations is insufficient).

Our third supplementary model restricts the sample to only those individuals who are interviewed in April (model 3) or May (model 4 and 5) for each year since 2015. In these models we have 4 observations in April/May before the Covid outbreak and 1 observation in April/May after the Covid outbreak, all taken at a year distance

Finally, while we worked most intensively on comparing yearly data, we also tried to compare monthly data, i.e. to compare those interviewed in the months after the outburst of Covid-19 to those interviewed in the months just before the outbreak however the number of observations in the first three months of 2020 is too small to allow us to do any analysis.

Overall any of the above mentioned feasible alternative specifications describe the same pattern, although the coefficients must be taken with a generous pinch of salt because of the small sample sizes.

**Fig 2:**
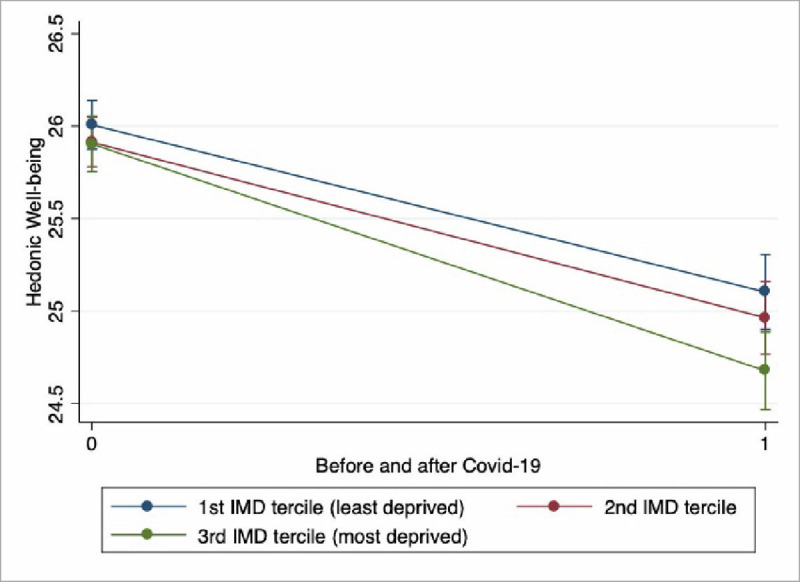
Change in Hedonic Well-being across the Covid-19 outburst by neighbourhood deprivation terciles. Authors computation on Understanding Society.

**Fig 3:**
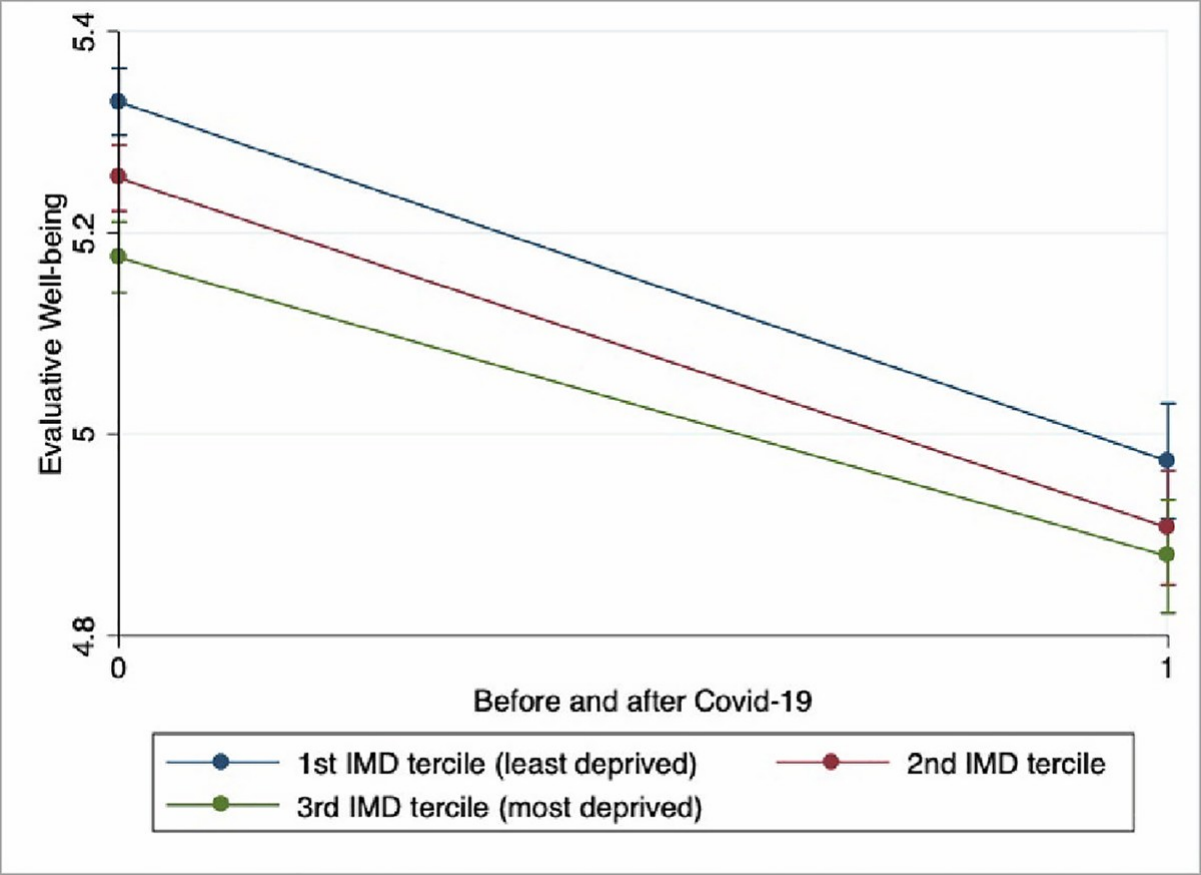
Change in Evaluative Well-being across the Covid-19 outburst by neighbourhood deprivation terciles. Authors computation on Understanding Society.

Figs 2 and 3 show that while the gap in hedonic well-being between individuals in the first and third deprivation terciles has widened significantly after the Covid-19 crisis, the same gap for evaluative well-being on the contrary has narrowed, although not significantly. This highlights that, as compared to individuals in less deprived areas, individuals in the most deprived ones have suffered much more in terms of hedonic well-being than in evaluative terms.

## Discussion

Since the beginning of the crisis, both levels of hedonic and evaluative well-being have decreased as a result of the pandemic and lockdown. However, for those living in more deprived neighbourhoods the level of hedonic well-being decreased more than for those living in better areas. We found no such difference for evaluative well-being.

Before the Covid-19 lockdown both hedonic and evaluative well-being measures were strongly negatively correlated with neighbourhood deprivation, but the restrictions imposed during the lockdown have had a different effect on the two correlations. While the negative effect of neighbourhood deprivation on hedonic well-being further strengthened during the lockdown, the negative effect of neighbourhood deprivation on evaluative well-being weakens and it is not statistically significant. This means that while the gap between those living in more or less deprived neighbourhoods has widened with respect to hedonic well-being, it has not significantly changed with regard to evaluative well-being.

At first glance, the differential effect of the lockdown on the relationship between neighbourhood deprivation and the different dimensions of well-being is surprising. While the type of questions and issues relating to hedonic well-being lead individuals to focus mainly on themselves and on reporting daily affective status, considerations of evaluative well-being often lead individuals to compare themselves and their previous situation to those of others. The information contributing to the measure of evaluative well-being provided by a sample of Canadians, for example, refer to one’s momentary affective state (only for 41 to 53 percent), by future expectations (22 to 40 percent), past events (5 to 20 percent), and social comparisons (5 to 13 percent) [15, 36]. Thus, in the case of an unexpected and tragic event such as the current Covid-19 crisis, individuals may experience a greater level of intense, short term, distress and negative feelings, but the crisis might be less relevant for the long-term. In fact, this is coherent with previous studies which analysed the effect of the pandemic on single GHQ questions, finding that the question with the largest deterioration was enjoyment of normal day-to-day activities [22].

There are various social and environmental features of socio-economic deprived neighbourhoods that can be responsible for decreasing hedonic well-being and mental health of individuals. The lack of green space, for example, is a relevant contributor to mental health deterioration [37]. Accordingly, in our sample the number of individuals that have mentioned access to outside space at home is around 20% higher for those living in the least deprived quintile than those in the most deprived quintile across the Covid-19 waves. Moreover, housing conditions are also likely to matter. Just before the outburst of the crisis, houses in the least deprived quintile of neighbourhood deprivation have on average two more rooms than those in the most deprived quintile. However, in the most deprived areas the average household size is 3.05 against 2.76 in the least deprived quintile. Therefore, conditions of relative overcrowding, where there are a greater number of people in a smaller house, can become significantly harder to bear in lockdown periods than in normal times. In addition to that, there is also evidence that the Covid-19 pandemic has been associated with lower social cohesion across the UK population [38]. Individuals in the most deprived areas, together with ethnic minorities and people under the age of 35, are those who have suffered the most from the decline in neighbouring relationship.

It is also interesting to further explore the reason behind the null effect of neighbourhood deprivation on evaluative well-being during the pandemic. In fact, while also evaluative well-being has overall decreased for both people living in more deprived and less deprived areas, the gap between them has remained stable and, if anything, has narrowed. In times of crisis, individuals can be more inclined to see their overall life situation in a different light, depending on the type of judgment they made and the extent to which they rely on social comparison with others [39]. Individuals residing in less deprived areas are likely to have more resources, both financial and inter-personal, than those in more deprived ones, and thus to possibly have “more to lose” in the context of a crisis. In our analysis, around 44% of the individuals who always worked from home during the lockdown lived in the two least deprived quintiles while over 50% of those who never worked from home in the same period lived in the two most deprived ones. It could be, thus, that those individuals who worked always from home were the ones who felt the greatest loss in terms of inter-personal resources, a potentially long-lasting loss if we think in terms of networking and future working opportunities.

Under this perspective, it is also interesting to consider individuals’ perceived control over their own life, frequently defined as self-efficacy. Neighbourhood deprivation tends to be associated with low levels of self-efficacy above and beyond individual SES [40], meaning that people living in more deprived areas tend to be more used to feel they do not have much control over their life, “a sense that one’s actions are chronically influenced by external forces outside of one’s individual control and influence” [41, p. 549]. Unpredictable events such as the outbreak of Covid-19 might, therefore, affect more negatively individuals living in less deprived neighbourhoods since they may experience a biggest disruption in their perceived ability to control their own life. Finally, a number of studies point to the relative exposure to Covid-19 news and knowledge [42]. For example in the US, African Americans, men and younger people have less accurate knowledge than white Americans, women and older individuals [43]. In our sample, we can see that more than 31% of low educated individuals lived in the most deprived quintile while around 50% of the high educated lived in the two least deprived quintiles. We could assume that individuals in more deprived neighbourhoods tend to have less exposure to Covid-19 news and knowledge. As less informed, such individuals might be worried about the long-term consequence of the crisis, but to a lower extent than those living in less deprived areas.

To sum up, our results highlight that both hedonic and evaluative well-being have decreased after the start of the pandemic but people living in the most deprived areas in the UK have seen their day-to-day well-being decrease much more than those living in better areas. Considered that even in the pre-Covid-19 world neighbourhood deprivation was negatively correlated with individual subjective well-being [9, 10], our results urge policymakers to rapidly act to diminish neighbourhood inequalities and reduce the increasing spatial clustering of disadvantage which has been enlarged by the pandemic.

## Supporting information

S1 Table Hedonic Well-being, base model, OLS cross-section by wave. (DOCX)

S2 Table Evaluative Well-being, base model, OLS cross-section by wave. (DOCX)

S3 Table Hedonic Well-being, individual controls, OLS cross-section by wave. (DOCX)

S4 Table Evaluative Well-being, individual controls, OLS cross-section by wave. (DOCX)

S5 Table Hedonic Well-being, individual and household controls, OLS cross-section by wave. (DOCX)

S6 Table Evaluative Well-being, individual and household controls, OLS cross-section by wave. (DOCX)

S7 Table Alternative models, robustness checks for the DID approach. (DOCX)
