# Supplementary material for: The combined effect of Covid-19 and neighbourhood deprivation on two dimensions of subjective well-being: Empirical evidence from England
Source: PLoS One. 2021 Jul 23;16(7):e0255156. doi: 10.1371/journal.pone.0255156 (PMC8301628; doi:10.1371/journal.pone.0255156)
Supplement: S1 Table — (DOCX) [file pone.0255156.s001.docx]

**S1 Table: Hedonic Well-being, base model, OLS cross-section by wave**

|  | Pre-Covid-19 | | | | Covid-19 | | | |
| --- | --- | --- | --- | --- | --- | --- | --- | --- |
| VARIABLES | -3 | -2 | -1 | 0 | 1 | 2 | 3 | 4 |
|  |  |  |  |  |  |  |  |  |
| **Neighbourhood deprivation** | -0.501*** | -0.521*** | -0.530*** | -0.496*** | -0.631*** | -0.945*** | -0.824*** | -0.599*** |
|  | (0.076) | (0.078) | (0.082) | (0.055) | (0.137) | (0.167) | (0.166) | (0.152) |
| Constant | 26.275*** | 25.986*** | 25.804*** | 25.695*** | 24.655*** | 24.547*** | 24.475*** | 25.191*** |
|  | (0.062) | (0.064) | (0.068) | (0.052) | (0.109) | (0.123) | (0.129) | (0.123) |
|  |  |  |  |  |  |  |  |  |
| Observations | 9,298 | 9,292 | 9,219 | 10,924 | 8,090 | 7,475 | 7,132 | 6,991 |
| R-squared | 0.009 | 0.009 | 0.009 | 0.008 | 0.012 | 0.027 | 0.019 | 0.012 |

Robust standard errors in parentheses. Weighted results. *** p<0.01, ** p<0.05, * p<0.1
