# Supplementary material for: The combined effect of Covid-19 and neighbourhood deprivation on two dimensions of subjective well-being: Empirical evidence from England
Source: PLoS One. 2021 Jul 23;16(7):e0255156. doi: 10.1371/journal.pone.0255156 (PMC8301628; doi:10.1371/journal.pone.0255156)
Supplement: S2 Table — (DOCX) [file pone.0255156.s002.docx]

**S2 Table: Evaluative Well-being, base model, OLS cross-section by wave**

|  | Pre-Covid-19 | | | | Covid-19 | |
| --- | --- | --- | --- | --- | --- | --- |
| VARIABLES | -3 | -2 | -1 | 0 | 2 | 4 |
|  |  |  |  |  |  |  |
| **Neighbourhood deprivation** | -0.185*** | -0.191*** | -0.212*** | -0.209*** | -0.167*** | -0.144*** |
|  | (0.019) | (0.020) | (0.020) | (0.014) | (0.034) | (0.039) |
| Constant | 5.336*** | 5.265*** | 5.189*** | 5.177*** | 4.753*** | 4.946*** |
|  | (0.016) | (0.017) | (0.017) | (0.013) | (0.031) | (0.030) |
|  |  |  |  |  |  |  |
| Observations | 9,320 | 9,313 | 9,261 | 10,974 | 7,500 | 7,016 |
| R-squared | 0.017 | 0.018 | 0.022 | 0.023 | 0.013 | 0.010 |

Robust standard errors in parentheses. Weighted results. *** p<0.01, ** p<0.05, * p<0.1
