# Supplementary material for: The combined effect of Covid-19 and neighbourhood deprivation on two dimensions of subjective well-being: Empirical evidence from England
Source: PLoS One. 2021 Jul 23;16(7):e0255156. doi: 10.1371/journal.pone.0255156 (PMC8301628; doi:10.1371/journal.pone.0255156)
Supplement: S3 Table — (DOCX) [file pone.0255156.s003.docx]

**S3 Table: Hedonic Well-being, individual controls, OLS cross-section by wave**

|  | Pre-Covid | | | | Covid | | | |
| --- | --- | --- | --- | --- | --- | --- | --- | --- |
| VARIABLES | -3 | -2 | -1 | 0 | 1 | 2 | 3 | 4 |
|  |  |  |  |  |  |  |  |  |
| **Neighbourhood deprivation** | **-0.084** | **-0.052** | **-0.067** | **-0.003** | **-0.115** | **-0.452***** | **-0.350**** | **-0.234*** |
|  | **(0.071)** | **(0.070)** | **(0.071)** | **(0.053)** | **(0.120)** | **(0.148)** | **(0.147)** | **(0.129)** |
|  | **Individual** | | | | | | | |
| Gender (female) | -1.030*** | -0.817*** | -0.926*** | -0.866*** | -1.764*** | -1.593*** | -1.582*** | -1.157*** |
|  | (0.115) | (0.117) | (0.123) | (0.096) | (0.201) | (0.234) | (0.239) | (0.218) |
| Age | 0.042*** | 0.043*** | 0.053*** | 0.051*** | 0.067*** | 0.056*** | 0.057*** | 0.056*** |
|  | (0.005) | (0.005) | (0.005) | (0.004) | (0.009) | (0.009) | (0.010) | (0.009) |
| Ethnicity (non-white) | 0.552** | 0.460* | 0.560** | 0.433*** | 0.265 | 0.694 | 0.579 | 1.093** |
|  | (0.237) | (0.236) | (0.241) | (0.157) | (0.474) | (0.551) | (0.511) | (0.448) |
| Medium education | 0.269 | 0.136 | -0.267 | -0.329 | -0.604 | -0.658 | -0.832 | -0.030 |
|  | (0.286) | (0.300) | (0.310) | (0.236) | (0.587) | (0.556) | (0.628) | (0.646) |
| High education | -0.001 | -0.187 | -0.517* | -0.435* | -1.067* | -1.095** | -1.132* | -0.531 |
|  | (0.285) | (0.299) | (0.308) | (0.233) | (0.576) | (0.543) | (0.615) | (0.615) |
| Other education | 0.484 | -0.104 | -0.508 | -0.329 | -0.986 | -1.178 | -1.119 | 0.010 |
|  | (0.332) | (0.347) | (0.372) | (0.279) | (0.725) | (0.744) | (0.744) | (0.670) |
| Mid financial security | 3.636*** | 4.000*** | 4.517*** | 3.790*** | 4.192*** | 4.362*** | 4.889*** | 6.190*** |
|  | (0.455) | (0.445) | (0.452) | (0.304) | (0.804) | (0.918) | (0.756) | (0.880) |
| High financial security | 5.587*** | 6.083*** | 6.638*** | 6.210*** | 7.229*** | 7.646*** | 6.630*** | 9.167*** |
|  | (0.428) | (0.414) | (0.425) | (0.284) | (0.748) | (0.844) | (0.706) | (0.813) |
| Mid financial security | -2.198*** | -2.063*** | -2.290*** | -2.343*** | -1.098*** | -0.890*** | -1.599*** | -0.919*** |
|  | (0.138) | (0.133) | (0.136) | (0.107) | (0.218) | (0.247) | (0.246) | (0.231) |
| Self-employed | 0.760*** | 0.383 | 0.208 | 0.688*** | 0.916 | -0.523 | 0.073 | 1.487 |
|  | (0.273) | (0.262) | (0.273) | (0.225) | (1.072) | (0.784) | (0.842) | (0.989) |
| Employee | 0.504** | 0.330 | -0.128 | 0.100 | 0.625 | -0.726 | -0.190 | 1.314 |
|  | (0.235) | (0.222) | (0.248) | (0.190) | (1.060) | (0.730) | (0.789) | (0.967) |
| Can work from home: sometime | -0.283 | -0.339** | -0.323* | -0.309** | -0.119 | -0.226 | -0.553 | -0.411 |
|  | (0.175) | (0.169) | (0.175) | (0.143) | (0.342) | (0.373) | (0.471) | (0.310) |
| Can work from home: always | -0.005 | 0.211 | -0.020 | -0.237 | -0.600** | -0.730** | -0.597* | -1.015*** |
|  | (0.279) | (0.276) | (0.303) | (0.244) | (0.274) | (0.310) | (0.330) | (0.279) |
| Living with a partner | 0.372** | 0.350** | 0.169 | 0.315*** | 0.504** | 0.662*** | 0.701** | 0.302 |
|  | (0.148) | (0.147) | (0.148) | (0.118) | (0.257) | (0.246) | (0.306) | (0.260) |
| Constant | 19.648*** | 19.301*** | 19.082*** | 19.088*** | 16.706*** | 17.888*** | 18.381*** | 14.559*** |
|  | (0.632) | (0.635) | (0.660) | (0.447) | (1.418) | (1.207) | (1.214) | (1.497) |
|  |  |  |  |  |  |  |  |  |
| Observations | 9,298 | 9,292 | 9,219 | 10,924 | 8,090 | 7,475 | 7,132 | 6,991 |
| R-squared | 0.136 | 0.154 | 0.190 | 0.184 | 0.182 | 0.188 | 0.165 | 0.214 |

Robust standard errors in parentheses; *** p<0.01, ** p<0.05, * p<0.1; Reference categories: Education (Low), Employment (Unemployed), Can work from home (Never), Financial security (Low). Weighted results.
