# Supplementary material for: The combined effect of Covid-19 and neighbourhood deprivation on two dimensions of subjective well-being: Empirical evidence from England
Source: PLoS One. 2021 Jul 23;16(7):e0255156. doi: 10.1371/journal.pone.0255156 (PMC8301628; doi:10.1371/journal.pone.0255156)
Supplement: S4 Table — (DOCX) [file pone.0255156.s004.docx]

**S4 Table: Evaluative Well-being, individual controls, OLS cross-section by wave**

|  | Pre-covid | | | | Covid | |
| --- | --- | --- | --- | --- | --- | --- |
| VARIABLES | -3 | -2 | -1 | 0 | 2 | 4 |
|  |  |  |  |  |  |  |
| **Neighbourhood deprivation** | **-0.059***** | **-0.052***** | **-0.078***** | **-0.058***** | **-0.050** | **-0.055** |
|  | **(0.018)** | **(0.018)** | **(0.019)** | **(0.013)** | **(0.031)** | **(0.034)** |
|  | **Individual** | | | | | |
| Gender (female) | 0.018 | -0.006 | 0.099*** | 0.005 | 0.006 | 0.035 |
|  | (0.030) | (0.031) | (0.032) | (0.024) | (0.062) | (0.059) |
| Age | 0.006*** | 0.006*** | 0.007*** | 0.005*** | 0.001 | -0.002 |
|  | (0.001) | (0.001) | (0.001) | (0.001) | (0.003) | (0.002) |
| Ethnicity (non-white) | -0.061 | -0.086 | -0.105* | -0.122*** | -0.001 | -0.091 |
|  | (0.062) | (0.059) | (0.062) | (0.038) | (0.106) | (0.112) |
| Medium education | 0.002 | -0.024 | -0.036 | -0.009 | -0.018 | -0.074 |
|  | (0.081) | (0.080) | (0.089) | (0.066) | (0.161) | (0.152) |
| High education | -0.016 | -0.011 | 0.006 | 0.053 | 0.107 | 0.000 |
|  | (0.080) | (0.079) | (0.088) | (0.066) | (0.161) | (0.151) |
| Other education | -0.012 | 0.066 | -0.013 | -0.042 | -0.182 | -0.113 |
|  | (0.098) | (0.094) | (0.102) | (0.078) | (0.223) | (0.190) |
| Mid financial security | 0.704*** | 0.907*** | 0.822*** | 0.833*** | 0.922*** | 0.965*** |
|  | (0.108) | (0.097) | (0.097) | (0.066) | (0.156) | (0.152) |
| High financial security | 1.470*** | 1.640*** | 1.571*** | 1.652*** | 1.540*** | 1.734*** |
|  | (0.101) | (0.088) | (0.089) | (0.061) | (0.151) | (0.136) |
| Mid financial security | -0.446*** | -0.485*** | -0.482*** | -0.493*** | -0.193*** | -0.238*** |
|  | (0.034) | (0.035) | (0.035) | (0.027) | (0.061) | (0.060) |
| Self-employed | -0.013 | 0.016 | 0.122* | 0.033 | -0.029 | 0.405* |
|  | (0.076) | (0.074) | (0.072) | (0.057) | (0.238) | (0.210) |
| Employee | 0.022 | -0.028 | -0.067 | -0.074 | -0.109 | 0.394* |
|  | (0.060) | (0.063) | (0.064) | (0.046) | (0.233) | (0.203) |
| Can work from home: sometime | -0.011 | 0.010 | -0.043 | 0.011 | 0.003 | -0.160 |
|  | (0.043) | (0.044) | (0.046) | (0.035) | (0.099) | (0.099) |
| Can work from home: always | 0.006 | 0.144** | -0.026 | 0.004 | -0.025 | -0.079 |
|  | (0.070) | (0.072) | (0.076) | (0.061) | (0.075) | (0.078) |
| Living with a partner | 0.238*** | 0.189*** | 0.181*** | 0.161*** | 0.319*** | 0.189*** |
|  | (0.038) | (0.037) | (0.039) | (0.030) | (0.069) | (0.066) |
| Constant | 3.723*** | 3.547*** | 3.542*** | 3.565*** | 3.317*** | 3.245*** |
|  | (0.159) | (0.149) | (0.156) | (0.107) | (0.331) | (0.295) |
|  |  |  |  |  |  |  |
| Observations | 9,320 | 9,313 | 9,261 | 10,974 | 7,500 | 7,016 |
| R-squared | 0.142 | 0.161 | 0.173 | 0.196 | 0.102 | 0.116 |

Robust standard errors in parentheses; *** p<0.01, ** p<0.05, * p<0.1; Reference categories: Education (Low), Employment (Unemployed), Can work from home (Never), Financial security (Low). Weighted results.
