# Supplementary material for: The combined effect of Covid-19 and neighbourhood deprivation on two dimensions of subjective well-being: Empirical evidence from England
Source: PLoS One. 2021 Jul 23;16(7):e0255156. doi: 10.1371/journal.pone.0255156 (PMC8301628; doi:10.1371/journal.pone.0255156)
Supplement: S5 Table — (DOCX) [file pone.0255156.s005.docx]

**S5 Table: Hedonic Well-being, individual and household controls, OLS cross-section by wave**

|  | Pre-Covid | | | | | Covid | | |
| --- | --- | --- | --- | --- | --- | --- | --- | --- |
| VARIABLES | -3 | -2 | -1 | 0 | 1 | 2 | 3 | 4 |
|  |  |  |  |  |  |  |  |  |
| Neighbourhood deprivation | -0.039 | 0.063 | 0.035 | 0.049 | -0.065 | -0.393*** | -0.280** | -0.174 |
|  | (0.076) | (0.073) | (0.077) | (0.055) | (0.119) | (0.144) | (0.142) | (0.129) |
|  | **Individual** | | | | | | | |
| Gender (female) | -1.016*** | -0.815*** | -0.926*** | -0.846*** | -1.740*** | -1.598*** | -1.514*** | -1.152*** |
|  | (0.114) | (0.117) | (0.122) | (0.096) | (0.202) | (0.230) | (0.235) | (0.219) |
| Age | 0.046*** | 0.047*** | 0.059*** | 0.058*** | 0.066*** | 0.063*** | 0.063*** | 0.056*** |
|  | (0.006) | (0.006) | (0.006) | (0.005) | (0.011) | (0.012) | (0.012) | (0.010) |
| Ethnicity (non-white) | 0.495** | 0.397* | 0.507** | 0.440*** | 0.236 | 0.548 | 0.409 | 1.035** |
|  | (0.237) | (0.236) | (0.244) | (0.161) | (0.470) | (0.531) | (0.506) | (0.467) |
| Medium education | 0.243 | 0.033 | -0.353 | -0.326 | -0.652 | -0.695 | -0.865 | -0.116 |
|  | (0.287) | (0.302) | (0.312) | (0.234) | (0.579) | (0.546) | (0.649) | (0.627) |
| High education | -0.011 | -0.282 | -0.667** | -0.433* | -1.100* | -1.084** | -1.116* | -0.612 |
|  | (0.290) | (0.307) | (0.313) | (0.235) | (0.573) | (0.534) | (0.641) | (0.607) |
| Other education | 0.463 | -0.151 | -0.525 | -0.325 | -0.957 | -1.182 | -1.137 | -0.063 |
|  | (0.333) | (0.348) | (0.372) | (0.277) | (0.723) | (0.736) | (0.763) | (0.652) |
| Mid financial security | 3.623*** | 3.883*** | 4.454*** | 3.769*** | 4.160*** | 4.138*** | 4.775*** | 6.172*** |
|  | (0.452) | (0.440) | (0.448) | (0.304) | (0.791) | (0.889) | (0.739) | (0.854) |
| High financial security | 5.551*** | 5.909*** | 6.519*** | 6.169*** | 7.072*** | 7.338*** | 6.463*** | 9.043*** |
|  | (0.431) | (0.413) | (0.426) | (0.287) | (0.737) | (0.833) | (0.699) | (0.784) |
| Underlying health condition | -2.158*** | -2.014*** | -2.226*** | -2.299*** | -1.102*** | -0.875*** | -1.583*** | -0.903*** |
|  | (0.136) | (0.133) | (0.135) | (0.107) | (0.217) | (0.242) | (0.236) | (0.225) |
| Self-employed | 0.721*** | 0.332 | 0.129 | 0.684*** | 0.867 | -0.459 | 0.375 | 1.379 |
|  | (0.274) | (0.261) | (0.271) | (0.224) | (1.033) | (0.805) | (0.877) | (0.980) |
| Employee | 0.510** | 0.340 | -0.201 | 0.138 | 0.617 | -0.595 | 0.141 | 1.260 |
|  | (0.237) | (0.221) | (0.244) | (0.190) | (1.024) | (0.760) | (0.840) | (0.962) |
| Can work from home: sometime | -0.284 | -0.380** | -0.403** | -0.341** | -0.090 | -0.225 | -0.528 | -0.442 |
|  | (0.176) | (0.171) | (0.176) | (0.144) | (0.336) | (0.375) | (0.459) | (0.309) |
| Can work from home: always | -0.012 | 0.156 | -0.104 | -0.260 | -0.595** | -0.706** | -0.573* | -1.031*** |
|  | (0.279) | (0.280) | (0.303) | (0.245) | (0.269) | (0.305) | (0.327) | (0.278) |
| Living with a partner | 0.254 | 0.119 | -0.095 | 0.200 | 0.481* | 0.432 | 0.592* | 0.272 |
|  | (0.162) | (0.157) | (0.163) | (0.129) | (0.288) | (0.287) | (0.335) | (0.289) |
|  | **Household** | | | | | | | |
| Private rent | 0.320 | -0.156 | 0.324 | 0.140 | -0.283 | -2.484 | 0.220 | 0.065 |
|  | (0.275) | (0.299) | (0.285) | (0.243) | (1.024) | (1.800) | (0.533) | (0.457) |
| Social rent | -0.615** | -1.282*** | -0.935*** | -0.896*** | -0.526 | -0.537 | -0.425 | -0.549 |
|  | (0.262) | (0.269) | (0.284) | (0.213) | (0.411) | (0.385) | (0.487) | (0.428) |
| Other tenure | -2.282 | 1.886** | -1.449 | -0.489 | -0.063 | -0.375 | 0.831 | -0.607 |
|  | (1.914) | (0.739) | (1.625) | (0.916) | (1.147) | (1.038) | (0.958) | (1.121) |
| Number of children in the hh | -0.073 | -0.014 | 0.128 | 0.126* | -0.357** | -0.387** | -0.661*** | -0.150 |
|  | (0.099) | (0.093) | (0.102) | (0.074) | (0.179) | (0.188) | (0.249) | (0.207) |
| Household size | 0.130* | 0.147** | 0.113 | 0.030 | 0.131 | 0.344** | 0.386** | 0.094 |
|  | (0.074) | (0.071) | (0.077) | (0.048) | (0.112) | (0.145) | (0.165) | (0.137) |
| Household earnings, med | -0.144 | -0.376** | 0.056 | -0.214 | -0.194 | -0.368 | -0.142 | -0.128 |
|  | (0.163) | (0.174) | (0.179) | (0.140) | (0.264) | (0.321) | (0.318) | (0.258) |
| Household earnings, high | -0.132 | -0.263 | 0.232 | -0.193 | -0.052 | -0.214 | 0.124 | -0.015 |
|  | (0.168) | (0.172) | (0.179) | (0.148) | (0.276) | (0.298) | (0.319) | (0.337) |
| Constant | 19.400*** | 19.511*** | 18.852*** | 18.891*** | 16.917*** | 17.328*** | 17.280*** | 14.710*** |
|  | (0.713) | (0.709) | (0.757) | (0.526) | (1.443) | (1.456) | (1.453) | (1.506) |
|  |  |  |  |  |  |  |  |  |
| Observations | 9,298 | 9,292 | 9,219 | 10,924 | 8,090 | 7,475 | 7,132 | 6,991 |
| R-squared | 0.139 | 0.161 | 0.195 | 0.187 | 0.184 | 0.193 | 0.173 | 0.217 |

Robust standard errors in parentheses; *** p<0.01, ** p<0.05, * p<0.1; Reference categories: Education (Low), Employment (Unemployed), Can work from home (Never), Financial security (Low), Tenure (Owned), Household earnings (Low), Household size goes from 1 to 14, number of children in the household ranges 0 to 7. Weighted results.
