# Supplementary material for: The combined effect of Covid-19 and neighbourhood deprivation on two dimensions of subjective well-being: Empirical evidence from England
Source: PLoS One. 2021 Jul 23;16(7):e0255156. doi: 10.1371/journal.pone.0255156 (PMC8301628; doi:10.1371/journal.pone.0255156)
Supplement: S6 Table — (DOCX) [file pone.0255156.s006.docx]

**S6 Table: Evaluative Well-being, individual and household controls, OLS cross-section by wave**

|  | Pre-Covid | | | | Covid | |
| --- | --- | --- | --- | --- | --- | --- |
| VARIABLES | -3 | -2 | -1 | 0 | 2 | 4 |
|  |  |  |  |  |  |  |
| Neighbourhood deprivation | -0.038** | -0.016 | -0.049*** | -0.029** | -0.027 | -0.044 |
|  | (0.019) | (0.019) | (0.019) | (0.014) | (0.031) | (0.035) |
|  | **Individual** | | | | | |
| Gender (female) | 0.020 | -0.004 | 0.097*** | 0.014 | 0.008 | 0.033 |
|  | (0.030) | (0.031) | (0.032) | (0.024) | (0.062) | (0.059) |
| Age | 0.006*** | 0.006*** | 0.008*** | 0.006*** | 0.001 | -0.000 |
|  | (0.002) | (0.002) | (0.002) | (0.001) | (0.003) | (0.003) |
| Ethnicity (non-white) | -0.063 | -0.088 | -0.117* | -0.099** | 0.001 | -0.107 |
|  | (0.062) | (0.060) | (0.064) | (0.040) | (0.106) | (0.114) |
| Medium education | -0.019 | -0.065 | -0.063 | -0.031 | -0.045 | -0.065 |
|  | (0.081) | (0.081) | (0.089) | (0.066) | (0.164) | (0.157) |
| High education | -0.047 | -0.062 | -0.035 | 0.015 | 0.068 | 0.005 |
|  | (0.082) | (0.081) | (0.089) | (0.066) | (0.165) | (0.156) |
| Other education | -0.025 | 0.043 | -0.018 | -0.054 | -0.193 | -0.095 |
|  | (0.099) | (0.095) | (0.102) | (0.078) | (0.226) | (0.195) |
| Mid financial security | 0.698*** | 0.870*** | 0.795*** | 0.813*** | 0.876*** | 0.969*** |
|  | (0.106) | (0.096) | (0.097) | (0.066) | (0.153) | (0.150) |
| High financial security | 1.443*** | 1.570*** | 1.534*** | 1.596*** | 1.457*** | 1.727*** |
|  | (0.101) | (0.089) | (0.090) | (0.062) | (0.154) | (0.141) |
| Underlying health condition | -0.431*** | -0.472*** | -0.464*** | -0.480*** | -0.182*** | -0.226*** |
|  | (0.034) | (0.034) | (0.034) | (0.027) | (0.060) | (0.059) |
| Self-employed | -0.027 | -0.000 | 0.106 | 0.031 | -0.048 | 0.384* |
|  | (0.076) | (0.074) | (0.072) | (0.057) | (0.245) | (0.206) |
| Employee | 0.018 | -0.034 | -0.080 | -0.071 | -0.119 | 0.382* |
|  | (0.060) | (0.062) | (0.064) | (0.046) | (0.240) | (0.200) |
| Can work from home: sometime | -0.023 | -0.004 | -0.066 | -0.010 | -0.016 | -0.183* |
|  | (0.043) | (0.044) | (0.046) | (0.035) | (0.099) | (0.098) |
| Can work from home: always | -0.009 | 0.127* | -0.044 | -0.013 | -0.050 | -0.097 |
|  | (0.070) | (0.072) | (0.076) | (0.061) | (0.074) | (0.078) |
| Living with a partner | 0.208*** | 0.143*** | 0.105** | 0.137*** | 0.272*** | 0.145** |
|  | (0.041) | (0.041) | (0.043) | (0.032) | (0.075) | (0.071) |
|  | **Household** | | | | | |
| Private rent | 0.019 | -0.051 | -0.052 | -0.058 | -0.324 | 0.024 |
|  | (0.072) | (0.079) | (0.077) | (0.059) | (0.273) | (0.112) |
| Social rent | -0.251*** | -0.358*** | -0.281*** | -0.273*** | -0.195* | -0.122 |
|  | (0.068) | (0.068) | (0.069) | (0.052) | (0.104) | (0.124) |
| Other tenure | -0.706* | 0.487*** | 0.354 | 0.150 | -0.438** | -0.073 |
|  | (0.365) | (0.175) | (0.397) | (0.195) | (0.201) | (0.226) |
| Number of children in the hh | 0.029 | -0.001 | 0.053** | 0.028 | 0.011 | 0.041 |
|  | (0.023) | (0.025) | (0.026) | (0.019) | (0.053) | (0.055) |
| Household size | -0.002 | 0.019 | 0.020 | -0.002 | 0.011 | 0.032 |
|  | (0.018) | (0.018) | (0.018) | (0.012) | (0.032) | (0.035) |
| Household earnings, med. | -0.045 | -0.041 | -0.012 | 0.015 | -0.042 | -0.066 |
|  | (0.042) | (0.044) | (0.045) | (0.035) | (0.080) | (0.083) |
| Household earnings, high | -0.011 | -0.002 | 0.015 | 0.078** | 0.055 | 0.049 |
|  | (0.045) | (0.046) | (0.046) | (0.037) | (0.072) | (0.086) |
| Constant | 3.833*** | 3.701*** | 3.571*** | 3.574*** | 3.495*** | 3.105*** |
|  | (0.174) | (0.169) | (0.177) | (0.125) | (0.377) | (0.348) |
|  |  |  |  |  |  |  |
| Observations | 9,320 | 9,313 | 9,261 | 10,974 | 7,500 | 7,016 |
| R-squared | 0.146 | 0.167 | 0.178 | 0.201 | 0.106 | 0.119 |

Robust standard errors in parentheses; *** p<0.01, ** p<0.05, * p<0.1; Reference categories: Education (Low), Employment (Unemployed), Can work from home (Never), Financial security (Low), Tenure (Owned), Household earnings (Low), Household size goes from 1 to 14, number of children
