# Supplementary material for: The combined effect of Covid-19 and neighbourhood deprivation on two dimensions of subjective well-being: Empirical evidence from England
Source: PLoS One. 2021 Jul 23;16(7):e0255156. doi: 10.1371/journal.pone.0255156 (PMC8301628; doi:10.1371/journal.pone.0255156)
Supplement: S7 Table — (DOCX) [file pone.0255156.s007.docx]

**S7 Table: Alternative models, robustness checks for the DID approach**

|  | (1) | (2) | (3) | (4) | (5) |
| --- | --- | --- | --- | --- | --- |
|  | One to One April-May | One to One April | Synthethic Yearly Hedonic | Synthethic Yearly Hedonic | Synthethic Yearly Evaluative |
| VARIABLES |  |  | April | May | May |
|  |  |  |  |  |  |
| **Covid#stdIMDscore** | **-4.658***** | **-3.687***** | **-2.220***** | **-0.249*** | **-0.0371** |
|  | **(1.551)** | **(0.983)** | **(0.508)** | **(0.760)** | **(0.208)** |
| Constant | 189.7 | 40.96 | 37.97 | 14.08 | 2.626 |
|  | (216.3) | (29.60) | (25.24) | (22.21) | (7.438) |
| Individual controls  Wave  Month | Yes  No  Yes | Yes  No  No | Yes  Yes  No | Yes  Yes  No | Yes  Yes  No |
| Observations | 129 | 94 | 138 | 120 | 120 |
| R-squared | 0.711 | 0.985 | 0.241 | 0.205 | 0.119 |
| Number of pidp | 110 | 87 | 28 | 24 | 24 |

Robust standard errors in parentheses. *** p<0.01, ** p<0.05, * p<0.1.The interaction is composed by the continuous standardised IMD and a dummy variable taking value 1 in the waves of full lockdown; Individual controls: Age, Education, Employment status, Financial security, Underlying health problems, Possibility to work from home, Presence of a partner, Number of children in the household, Household size, Household earnings.
